# Assessment of factors influencing hygiene behaviour among school children in Mereb-Leke District, Northern Ethiopia: a cross-sectional study

**DOI:** 10.1186/1471-2458-14-1000

**Published:** 2014-09-26

**Authors:** Mulubirhan Assefa, Abera Kumie

**Affiliations:** Department of Public Health, College of health science Jigjiga University, Jigjiga, Ethiopia; Department of preventive medicine, School of Public health, Addis Ababa University, Addis Ababa, Ethiopia

**Keywords:** Hygiene, Behaviour, Factor-influencing, School children, Ethiopia

## Abstract

**Background:**

Poor school sanitation and hygiene is a major problem in developing countries and remains high risk behaviour among primary school going children. Many outbreaks of gastrointestinal infections have been associated with primary schools. This research paper was designed to assess the factors influencing hygiene behaviour among school children.

**Methods:**

A cross sectional study was conducted in Mereb-Leke District, Tigray National Regional State among school children. The study population consisted of those who are in the second cycle as they are more mature and most senior in primary schools. A multi-stage probability sampling procedure with three stages was used to select participated schools. A total of 528 school children were randomly selected from students networking list of selected schools. Structured questionnaire and observational checklist at home and school setting were used to collect data.

Statistical analysis was done using SPSS Version 17.0 after the data has been entered using Epi-Info version 3.5.3. Primarily variables that had p-value <0.2 at bivariate analysis were used to develop logistic model to identify factors influencing hygiene behaviour via crude and adjusted odds ratio.

**Results:**

Children were grouped according to whether positive or negative hygiene behaviour outcome which permitted identifying factor affecting hygiene behaviour. Out of these, 326 (61.7%) had positive hygiene behaviour. The study found that knowledge s on water handling (AOR, 2.24; 95% CI 1.54, 3.26), hand washing (AOR, 1.70; 95% CI 1.12, 2.57) and awareness on water handling matters (AOR, 2.0; 95% CI 1.37, 2.90), hand washing practice (AOR, 2.36; 95% CI 1.62, 3.45) were significantly associated to hygiene behaviour status.

Being a member of hygiene and sanitation club (COR 0.42; 95% CI 0.26, 0.68), parent’s health package status (COR 0.62; 95% CI 0.43, 0.90), training on hygiene and sanitation and experience of visiting model school (COR 1.99; 95% CI 1.37, 2.88) had significance difference in hygiene behaviour.

**Conclusion:**

This study has shown that knowledge, awareness, training on hygiene and sanitation, being a member of hygiene and sanitation club, experience of visiting model school, and parent’s health package status were factors influenced hygiene behaviour.

**Electronic supplementary material:**

The online version of this article (doi:10.1186/1471-2458-14-1000) contains supplementary material, which is available to authorized users.

## Background

Impact on disease burden due to inadequate and unsafe water, lack of sanitation and poor hygiene behaviour is a complex issue [1]. The occurrence and severity of Hygiene related outbreaks in endemic areas is greatly enhanced by human behaviour with regards the practice of healthy hygiene [2–4]. Poor hygiene behaviour is a major problem in developing countries [5]. Hygiene and sanitation related Diseases are a huge burden in developing countries; Causing many people to fall ill even to die [2], Schools have repeatedly been implicated in the spread of gastrointestinal disease, High among primary school going children [5–9].

Improvements in hygiene behaviour are the most important barrier to many infectious diseases, because with safe behaviour and appropriate facilities, people reduce their risk of becoming exposed to diseases [1–5]. A study conducted by FEACHEM R. G stated that hygiene behaviour influences the pattern of diarrhoeal spread. Water handling, latrine utilization behaviour, and hand-washing were the specific behaviour received most attention [10]. Among children for whom mainly positive hygiene behaviour was recorded, the prevalence of diarrhoea was 6.4 days per child-year, while it was 14.2 days per child year in children with mainly negative scores [11].

As hands is an important mode of transmission of infectious disease among school-aged children. Simple hand washing with soap helps to protect children from the two common global paediatric killers (diarrhoea and lower respiratory infection) [12–14], hand hygiene significantly reduce illness-related absences in elementary school students by 26% [15]. Critical times for hand washing include after using the toilet, after cleaning a child, and before handling food [16, 17].

The mere provision of water supply and sanitation facilities is not enough to bring down morbidity and mortality rates [2]. Water and sanitation facilities linked with hygiene behaviour have proven to be more effective in reducing diarrhoeal diseases [5, 6, 18] and to support the improvements of sustained behavioural change [12, 18, 19].

Attitudes, knowledge, and beliefs are some of the measures which are thought to be on the causal pathway to behaviour. Poor knowledge and practice of, and attitudes to personal hygiene has negative consequences for a child’s long term overall development [16]. A study conducted in Ethiopia found that 60% of children surveyed did not know about the possible transmission of diseases through human waste [20]. Awareness of health aspects of sanitation behaviour is important because it determines the degree of sustainability of an intervention in sanitation. Perception strongly influences one’s hand washing beliefs and practices.

The hygiene behaviour that children learn at school made possible through sanitation and hygiene-enabling facilities [9], and play a major role in ensuring good hand washing practices [12]. A study conducted in Ghana indicates that lack of hygiene enabling facilities at schools and homes did not allow children’s’ to practice the hand washing knowledge they had acquired [14].

Hand washing-facilities must be easily accessible and available at all times with the right materials necessary to make the process a success. A study conducted by Oswald and his Colleagues revealed that Lack of resources, namely soap and water, as well as inadequate sanitation facilities may be two of the main reasons why children do not wash their hands [21]. The location of hand washing led to some pupils forgetting to wash hands [22].

The family seemed to play a 50% of positive reinforcement compared to 27.3% who identified the school as a motivator [6]. At school setting teachers act as role models; they also provide leadership in hygiene related issues within the school [22]. Based on the study conducted in sub-Saharan Africa, motivating factors behind proper hand washing included avoidance of dirt and smell of defecation, stay healthy, clean people are more accepted, cleanliness is associated with better socioeconomic status, hands feel and smell fresh, and avoid the risk of disease [23]. Also, if the children had clean hands, they would have clean books; resulting in better grades [2–4].

District health office report of last 3 years (2010, 2011, 2012) showed that Diarrhoea, Intestinal parasites, Upper respiratory Infection were among the top five diseases of the district. Literatures show that Poor hygiene behaviour remains high risk behaviour increasingly responsible for high burden of these diseases [5, 6]. School children based research on Hygiene behaviour is required.

The factors which may determine hygiene behaviour among school children are complex, interlinked and some are difficult to measure.

Previous studies conducted in Ethiopia, particularly in the study area, provide limited details about factors that determine hygiene behaviour among school children. This study, therefore, had investigated factors influence hygiene behaviour among school children. The study bridges the information gap on school Hygiene behaviour and to set evidence based intervention at school setting.

## Method

### Study design

A school based cross-sectional study was conducted in Mereb-Leke District, Northern Ethiopia from July, 2012 to Jun, 2013. All children of primary school going age in Mereb-Leke district were considered as source population while the study populations were those who are attending second cycle education.

### Sample size estimation and sampling technique

The target participants for this study were school children in second cycle of every selected primary school in Mereb-Leke District. Five hundred twenty eight school children were estimated using two population proportion formulas to participate on the study and Epi-Info version 7 was used for sample size calculation.

A multi-stage probability sample procedure with three stages (district primary school; five selected primary schools; students networking list) was used to select five primary schools. They were selected randomly from the list of primary schools that have second cycle (20 schools) in the district Education Office. The reason for the choice of school children in second cycle (grade six to eight) is because they are the more mature and most senior in primary school.

A total of 528 school children were randomly selected from students networking list of selected school, based on the proportional allocation. Of whom, 264 student’s household,who have an even ID number, was selected using lottery method for further studying on hygiene enabling facilities at household level.

### Data collection

Structured questionnaire and observational checklist at home and school setting was used to collect data. The questionnaire was initially drafted in English, translated to Tigrigna, and then back to English.

Five teachers as data collection facilitator at school setting and six health extension workers as data collectors (house hold hygiene enabling facility) were recruited to facilitate and guide the data collection process. These study staff was given training for two days by the principal investigator on the objective of the study, techniques of assisting study participants whenever they come across difficulties in completing the questionnaire, in order to avoid incompleteness of the questionnaire. Then, the instrument was pre-tested on 28 students in a similar primary school in the study area which was not included in the study. The pre-test had conducted prior to the actual data collection time to assess the suitability of the questionnaire with regards to duration, language appropriateness, content, validity, and question comprehensibility. Some amendments were made after the pretest.

WHO indicated that Water handling, latrine utilization, and hand-washing are three key hygiene behaviors [5]. The questionnaire had consisted of: demographic information (gender, age); parent’s educational status (illiterate, primary/secondary, tertiary and more); parents’ health package status (graduated, not graduated but participating, not participating). Knowledge questions on the three key hygiene behaviour were assessed. The questionnaire includes awareness questions that were determining whether the school children believe that hygiene behaviour (hand washing, use and cleaning of toilet, handling drinking water) can actually help diseases prevention.(see Additional file 1: annex).

All questionnaires and records were checked for completeness by the data collectors and supervisors before leaving the area where data collection was done. The households’ hygiene enabling facility was cross checked for its accuracy and completeness by supervisor and principal investigator.

### Measurement used

#### Behaviour

Is “what a child does” and which is observable and measurable. Scores were assigned to the variables for stated behaviour using the issue scores as mentioned below; Score issue is hygiene behaviour: 0- no/never, 1-yes/always.

Hygiene behaviour is measured as a composite score for students who answered yes/always to at least 9 of fourteen questions requesting about practice and/or skill of water handling, latrine utilization, and hand washing was classified as having positive hygiene behaviour.

### Data analysis

After the data were entered in to Epi-Info version 3.5.3, a statistical analysis was done using SPSS Version 17.0. Descriptive analysis has done by calculating frequencies (response rate) of the knowledge, awareness (perception), and skill (practical) questions. The importance of knowledge, awareness, enabling factor as determinant of hand washing, latrine usage and keeping drinking water free from faecal contamination was also analysed. Data on the level of respondent’s knowledge, perception was compared with what they stated about their hygiene practice.

Moreover, logistic regression was employed to identify factors influencing hygiene behaviour via crude and adjusted odds ratio. Primarily variables that has p-value <0.2 at bivariate analysis were used to develop the logistic model in order to identify predisposing factors which more strongly linked with the hygiene behaviour outcome. Further general and specific (sex based, key behaviour based) analysis was also premeditated with respect to motivational/supportive factor (such as parents educational and health packages status), availability and accessibility of hygiene enabling facilities.

### Ethical consideration

Before commencement of the actual activities, ethical clearance was obtained from Addis Ababa University, School of public health ethical review committee. A written permission of the District education office was obtained and a letter of support was written to all respective head of the selected schools. The purpose of the study as well as its confidentiality of the information obtained (assign unique identification code) was entirely explained to school director. Then additional informed verbal consent was obtained from school director on behalf of the school children. In addition, a verbal consent has obtained from household head for the observation made at household level.

After the data collection done, a hand book designed for families health package by Ministry of Health was disseminated for each student participated in the study. The hand book has explained importance of proper hygiene on diarrhoeal prevention.

## Results

A total of five hundred twenty eight school children, were recruited from five primary schools, were participated in the study giving a response rate of 100%. The study revealed that 278 (52.5%) of the respondent was females and the mean age of the participants was 14.5 years. Out of the total, 164 (31%), 181 (34.3%), and 183 (34.7%) was grade six, seven, eight respectively. Most of the respondents, 304 (57.6%), were from urban and the remaining was from rural.

According to the criteria defined in the method part, Children were grouped whether positive or negative hygiene behaviour outcome which permits to identify factor affecting hygiene behaviour. Out of these, 326 (61.7%) had positive hygiene behaviour while 202 (38.3%) had negative hygiene behaviour.

### Predisposing factors influencing hygiene behaviour

#### Knowledge

Out of the total study participants 65% had adequate knowledge on water handling but more than 91.1% do not have proper knowledge on latrine utilization and 71% had no adequate knowledge on hand washing. Of those school children who had adequate knowledge on hand washing, water handling, and latrine utilization; 71.1%, 68.8%, and 53.2% was with positive hygiene behaviour respectively (Table 1).

**Table 1:** **The frequency of knowledge and awareness of school children in Mereb-Leke District, Northern Ethiopia March 2013**

Characteristics	Frequency (n = 528)	Percentage (%)
Knowledge on water handling		
Know	343	65
Don’t know	185	35
Knowledge on latrine utilization		
Know	47	8.9
Don’t know	487	91.1
Knowledge on hand washing		
Know	149	28.2
Don’t know	379	71.8
Awareness of water handling		
Aware	278	52.7
Not aware	250	47.3
Awareness of latrine utilization		
Aware	103	19.5
Not aware	425	80.5
Awareness of hand washing		
Aware	311	58.9
Not aware	217	41.1

### Awareness

The study revealed that more than half of the children were aware on hand washing and water handling accounts for 58.9% and 52.7%, respectively. The majority, however, 80.5% of the respondent was reported not aware to latrine utilization.

Among those who have awareness about water handling 71.6% had practiced positive hygiene behaviour and while 50.8% of those not aware had reported positive hygiene behaviour. According to the study a 24% difference in positive hygiene behaviour is the difference between those who have awareness of hand washing and those who do not have or what (Table 2).

**Table 2:** **Final logistic regression of predisposing factors influencing hygiene behaviour among school children in Mereb-Leke district, Tigray Region, Ethiopia March 2013**

Characteristic(s)	Frequency of hygiene behaviour (n = 528)		Crude OR (CI 95%)	Adjusted OR (CI 95%)
	Positive	Negative		
Knowledge on water handling				
Yes	236	107	2.33(1.61, 3.36)**	2.24(1.54, 3.26)**
No^#^	90	95	1	
Knowledge on latrine utilization				
Yes	25	22	0.68(0.37, 1.24)	0.85(0.46, 1.58)
No^#^	301	180	1	
Knowledge on hand washing				
Yes	106	43	1.78(1.18, 2.68)*	1.70(1.12, 2.57)*
No^#^	220	159	1	
Awareness for water handling				
Yes	199	79	2.44(1.70, 3.50)**	2.0(1.37, 2.90)**
No^#^	127	123	1	
Awareness for latrine utilization				
Yes	55	48	0.65(0.42, 1.01)	0.88(0.55, 1.40)
No^#^	271	154	1	
Awareness for hand washing				
Yes	223	88	2.81(1.95, 4.03)**	2.36(1.62, 3.45)**
No^#^	103	114	1	

^#^Reference group, P < 0.05*; P < 0.01**.

### Practice/skill

Respondents were asked if they treat their drinking water, overall 92.8% of the respondent reported that ‘yes’. The study indicated that 75.2% of the respondent had reported they have ever cleaning and covering water container but 42.2% of the study subject reported never touch drinking water by dirty hand. Out of those who boiled their drinking water, 67.8% (N = 242) of the student reported that they boiled their drinking water the day prior to data collection.

Among the school children 385 (73%) was reported to defecate in latrine and Out of those, 206 (53.6%) of the participant reported always to ‘how frequent use latrine’. Also, this study shows 272 (70.6%) of the respondent reported to execrate in latrine the day prior to data collection.

Of the school children, more than (370) 70% were not washing their hands after defecation and after eating. Even 463 (87.7%) reported they usually wash hands and 450(85.2%) wash their hands the day prior the data collection, 513 (97.2%) of the school children reported that they did not use soap at critical time. Besides, the study reports that 412 (78%) of the participants they didn’t practice the correct procedure of hand washing (Table 2).

Primarily variables that had p-value <0.2 at bivariate analysis were used to develop logistic in order to identify predisposing factors which more strongly linked with the hygiene behaviour outcome. On multivariate logistic regression knowledge, awareness was found to be significantly associated to hygiene behaviour.

The likelihood that a child who is knowledgeable on water handling issues 2.24 times (AOR, 2.24; 95% CI 1.54, 3.26) is more likely to have positive hygiene behaviour compare those who are not knowledgeable while hand washing matters 1.7times (AOR, 1.70; 95% CI 1.12, 2.57) more likely. Predictably, the reported awareness level of key behaviour was closely related to the hygiene behaviour outcome. School children who had proper awareness water handling matters (AOR, 2.0; 95% CI 1.37, 2.90), hand washing practice (AOR, 2.36; 95% CI 1.62, 3.45) were more likely to have positive hygiene behaviour compare to those school children not aware.

### Hygiene enabling factors influencing hygiene behaviour

As shown on Table 2, Out of the total five-hundred twenty eight respondents, (264)50% of study subject households were selected for assessing hygiene enabling factors (Table 3).

**Table 3:** **Distribution of household hygiene enabling facility in Mereb-Leke District, Tigray Region, Ethiopia March 2013**

House hold enabling facility	Frequency (n = 264)	Percentage %
Main source of drinking water		
Protected	174	65.9
Not protected	90	34.1
Time spent for one trip		
< 30 minutes	193	73.1
>30 minute	71	26.9
Drinking water storage		
Narrow necked	142	53.8
Wide	122	46.2
Cover with lid		
Yes	197	74.6
No	67	25.4
Separate cub next to container		
Yes	124	47
No	140	53
Latrine availability		
Yes	155	58.7
No	109	41.3
Recommended Physical structure of latrine**		
Yes	81	52.3
No	74	47.7
Cleanliness of the floor		
Clean	72	46.5
Not clean	83	53.5
Cleanliness of latrine**		
Good	47	30.3
Not good	108	69.7
Obstacles in the path**		
Yes	36	23.2
No	119	76.8
Depth of faeces to slab**		
<50 cm	57	36.8
>50 cm	98	63.2
Availability of hand washing facility		
Yes	145	55.3
No	119	44.7
Location of hand washing facility Near to latrine**		
Yes	54	37.2
No	91	62.8
Presence of soap at the facility**		
Yes	81	55.9
No	64	44.1
Where the family member wash their hands		
In the facility	145	54.9
Elsewhere	119	45.1

**Missing data excluded.

The study also showed that all, the five schools, had latrine facilities which were located within the school compound and all had gender segregated compartments for both students and staffs. Of the five schools, 4 schools had access to improved water source; but no one had functional hand washing facilities. The observation of each school reported that on average one seat serves for 73 students for boys, 80 for girls.

Of the school children, 35.4% and 37.3% reported that ‘always’ for the question is toilet paper available?’ and ‘cleanliness of toilet’, respectively. The study revealed that more than 47% of the respondent was reported ‘always’ to whether ‘queuing’ for using the latrine during break. 66.3% of 528 school children in the study delighted that they use soap and water to wash their hands while 33.7% needed to habit water only.

### Motivational/supportive factors

Among those students with positive hygiene behaviour (N = 326), 59% their mother and 27% of their father were unable to read and write. 60.7% of their parents were graduate and/or involved in the health package and 64.4% were farmers (Table 4).

**Table 4:** **Distribution of respondent’s hygiene behaviour outcome by motivational factors in Mereb-Leke District, Tigray Region, Ethiopia March 2013**

Characteristic	Hygiene behaviour outcome	
	Positive (%)	Negative (%)
Mother educational status:		
Unable read and write	192 (64.6)	105 (35.4)
Primary/secondary/complete and above	134 (58)	97 (42)
Father educational status:		
Unable read and write	88 (60.3)	58 (39.7)
Primary/secondary/complete and above	238 (62.3)	144 (37.7)
Occupational status:		
Farmer	210 (65.6)	110 (34.4)
Merchants and gov’t employees	116 (55.8)	92 (44.2)
Parents health package:		
Graduated and/or involved	198 (61.7)	123 (38.3)
Neither graduated nor involved	128 (61.8)	79 (38.2)
Training hygiene and sanitation:		
Yes	158 (60.3)	104 (39.7)
No	168 (63.2)	98 (36.8)
Visit model school:		
Yes	129 (62)	79 (38)
No	197 (61.6)	123 (38.4)
Hygiene and sanitation membership:		
Yes	167 (61.2)	106 (38.8)
No	125 (61.6)	78 (38.4)

Being a member of hygiene and sanitation club had observed a significance difference to water handling practice (COR 0.42; 95% CI 0.26, 0.68). The study revealed that a significance difference in hand washing behaviour was associated with parent’s health package (COR 0.62; 95% CI 0.43, 0.90), have ever trained on hygiene and sanitation (COR 1.99; 95% CI 1.37, 2.88), and have ever visit model school (COR 1.73; 95% CI 1.18, 2.54).

Out of the participants, 294(55.7%) had reported self-initiation for the question stating ‘who motivates to use latrine’ ; 246(46.6%) of the respondents was reported a reason of separate toilet for boys and girls to ‘what promote you to go to school latrine’.

## Discussion

Children are “agent of change” in pacing the behaviour and practice of their family and community at large. The determinant of hygiene behaviours’ of school children was inadequately studied in Ethiopia. In this study, the analysis and interpretation of the findings by comparing the key hygiene behaviour outcomes among school children provided a better understanding of the factors that influence hygiene behaviours.

Knowledge and awareness are some of the measures which are thought to be on the causal pathway to behaviour [1, 18]. According to the present study knowledge of students’ was gauged and the proportion of positive hygiene behaviour among school children was fairly high in those who had adequate knowledge. Result from logistic regression analysis found that a difference in hygiene behaviour outcome was statistically significant with knowledge status of the students on water handling issues (P < 0.001) and hand washing matters (P < 0.05). Accordingly, knowledge is important factor to have observed positive hygiene behaviour. Our study is consistent to the study conducted in India Chitungwiza, and Cameroon which indicated with improvement in knowledge level, respondent’s exhibit better hygiene practices [1, 3, 6].

Awareness of health aspects of sanitation behaviour is important because it determines the degree of sustainability of an intervention in sanitation [19]. In this study, a considerable gap has been observed between those who has good awareness and exhibit positive hygiene behaviour verses those who do not have both. Among those who aware to water handling 71.6% had practiced positive hygiene behaviour and 24% difference in positive hygiene behaviour was shown among school children due the awareness of hand washing. The overall awareness level was significantly associated with hygiene behaviour (P < 0.001). Though a large proportion of positive hygiene behaviour was associated with awareness, the multivariate analysis suggested that the awareness of latrine utilization did not play an important role in determining both latrine utilization practice and positive hygiene behaviour. The difference (high positive behaviour and low awareness) may in part be related to the fact that some hygiene behaviours are customary, being sustained as usual practices for traditional reasons, not necessarily related to hygiene awareness [1].

The hygiene behaviour that children learn made possible through hygiene-enabling facilities [9]. We have assessed the hygiene enabling facilities both at home and school setting using structured observational checklist. The study suggests that the proportion of having hygiene enabling facilities has shown difference in the key hygiene behaviour. The crude analysis suggested that self-reporting of queuing during break time play important role in determining the frequency of latrine usage. However, the logistic regression suggested that hygiene enabling factors did not play an important role in determining positive hygiene practices. On the contrary, a study conducted in Chitungwiza, Senegal and Peruvian shanty town shown that the hygiene enabling factor has a determinant role for positive hygiene behaviour [6, 21, 23]. Yet, Small sample size of the house hold may be one of the several potential reasons for these unexpected results. In the other the study shows that the behaviour of never touching drinking water by dirty hands was arise due to inaccessibility and unavailability to main source of drinking water.

Based on an understanding of the factors that influence hygiene behaviour; assessment of the motivational factor was addressed in this study. The large proportion of male student (82.5%) and female students (59.6%) had reported proper water handling practice and hand washing at critical time, respectively.

The motivational logistic regression suggested that the difference found in male student was significantly associated to have ever trained (P < 0.05) and being membership of hygiene and sanitation club (P < 0.02). The study also found that the proportion difference observed in female student was associated with visiting model school. Moreover, this study indicated that parent’s health package status and being trained on hygiene and sanitation were important in determining hand washing behaviour at P <0.02.

The present study revealed that teacher was found as motivator to initiate latrine utilization with a P-value of less than 0.05. Similarly, literature reviewed by Ana Gil indicates Teachers act as role models for school children. The peer pressure measured by ‘what you think children are washing their hands’ was observed a difference in hand washing behaviour and this was statistically significant with diseases avoidance at P <0.05.

## Conclusions

The study was carried out to assess the factors influencing hygiene behaviour through giving undistinguishable consideration to the three key hygiene behaviours. Knowledge and awareness on Water handling and hand washing are important predisposing factor to influence hygiene behaviour and Students observing considerable gap of positive hygiene behaviour.

Based on an understanding of the factors that influence hygiene behaviour; the assessment shown that motivational factor is significantly associated with hygiene behaviour issues. Training for male students, being a member to hygiene and sanitation club, experience of visiting model school, and parent’s health package has found associated to hygiene behaviour issues of the student.

## Authors’ information

Mulubirhan Assefa Alemayoh: BSc in Environmental Health Science, MPH in Epidemiology, Lecturer at Jigjiga University. Dr Abera Kumie Takele: MD, MSC, PhD, Associate Professor at Addis Ababa University/School of public health.

## Electronic supplementary material

Additional file 1: **Annexes of data collection tools.** House hold based questionnaire (interview and observation). (DOCX 46 KB)
