# Supplementary material for: Assessment of factors influencing hygiene behaviour among school children in Mereb-Leke District, Northern Ethiopia: a cross-sectional study
Source: BMC Public Health. 2014 Sep 26;14:1000. doi: 10.1186/1471-2458-14-1000 (PMC4190334; doi:10.1186/1471-2458-14-1000)
Supplement: Supplementary file 1 — Additional file 1: Annexes of data collection tools. House hold based questionnaire (interview and observation). (DOCX 46 KB) [file 12889_2014_7117_MOESM1_ESM.docx]

**Annexes of data collection tools**

**Self-administered questionnaire for school children**

**Student Identification Code: ___/___/___ , School identification code: ___/__/__**

| No | Questions | Possible answer | Skip To |
| --- | --- | --- | --- |
| 101 | sex | 1. Male 2. Female |  |
| 102 | Your grade | _____ |  |
| 103 | Age |  |  |
| 104 | Where did you live | 1. Urban 2. Rural |  |
| 105 | What is the Mothers educational status | 1. Unable to read and write 2. Primary/secondary complete 3. College and above |  |
| 106 | What is the Fathers educational status | 1. Unable to read and write 2. Primary/secondary complete 3. College and above |  |
| 107 | What is the Parents occupational status | 1. Farmer 2. Merchant 3. Government Employees 4. If Others, specify it |  |
| 108 | What is the Parents health package status | 1. Graduated 2. Involved 3. Neither graduated nor involved 4. Don’t know |  |
| 109 | Have ever trained in hygiene and sanitation | 1. Yes 2. No |  |
| 110 | Have you visit model school in your surrounding | 1. Yes 2. No |  |
| 111 | Is there hygiene and sanitation club in your school | 1. Yes 2. No 3. Don’t know | 113-If your Ans. is 2 or 3 |
| 112 | If yes, Are you a member | 1. Yes 2. No |  |
| 113 | Have you miss a class in previous two weeks | 1. Yes 2. No | 115- If your Ans. Is 2 |
| 114 | If yes, why you miss | 1. Sickness from communicable diseases 2. Family sickness 3. Reason other than illness | 115- If your Ans. Is 3 |
| 115 | Is the sickness from diarrhoea and /or water borne disease | 1. Yes 2. No |  |
| 116 | Do you know practicing hygiene activities reduce incidence of diarrhoeal disease | 1. Yes 2. No 3. Uncertain |  |
| **Water handling behaviour** | | | |
| 201 | Do you know that unclean/insanitary water can have health problem | 1. Yes 2. No 3. Don’t know |  |
| 202 | Do you know drinking water should never be touched as your hands have dirty | 1. Yes 2. No 3. Don’t know |  |
| 203 | Do you know Boiling water kills germs | 1. Yes 2. No 3. Don’t know |  |
| 204 | Did you know Water container needs cleaning and covering | 1. Yes 2. No 3. Don’t know |  |
| 205 | Do you think that keeping drinking water free from faecal contamination is important for preventing diarrhoea | 1. Yes 2. No 3. Uncertain |  |
| 206 | Do you think that treating water in any way to make it safer to drink is important | 1. Yes 2. No 3. Uncertain |  |
| 207 | What do you usually do to the water to make it safer to drink | 1. Boil 2. Add bleach/chlorine 3. Use a water filter | If 2 or 3 skip to 209 |
| 208 | Have Boiled drinking water yesterday | 1. Yes 2. No |  |
| 209 | Have you ever cleaned and cover water container | 1. Always 2. Sometimes 3. Never |  |
| 210 | Have you touched drinking water as your hands have dirty | 1. Yes 2. No |  |
| **Latrine utilization behaviour** | | | |
| 301 | Do you know Human faces contain germs**:** | 1. Yes 2. No 3. Don’t know |  |
| 302 | Can germs be transmitted from toilets by direct contact with or indirectly contaminated hands, or via insects | 1. Yes 2. No 3. Uncertain |  |
| 303 | Do you know importance of proper toilet usage for preventing diarrhoea | 1. Yes 2. No 3. Don’t know |  |
| 304 | Do you think proper latrine usage is better than getting medication for diarrhoea | 1. Yes 2. No 3. Uncertain |  |
| 305 | Do you think Open defecation may cause germs to spread | 1. Yes 2. No 3. Uncertain |  |
| 306 | Is a latrine available at school | 1. Yes 2. No | If 2 skip to 314 |
| 307 | have you use the school toilet for a ‘poo’ | 1. Usually 2. Only if desperate 3. Never |  |
| 308 | Who Motivates for using the toilets? (multiple response is possible) | 1. Self-initiation, 2. Parents 3. Peer pressure, 4. Teacher 5. Others, Specify |  |
| 309 | What assists you to go the toilet without coming contaminating school compounds  (multiple response is possible) | 1. Separate toilets for boys and girls 2. Cleanliness of toilet 3. Toilet privacy 4. Safety 5. Others,_________ |  |
| 310 | Are you ever bully in the school toilets | 1. Always 2. Sometimes 3. Never |  |
| 311 | Is there toilet paper available; | 1. Always 2. Sometimes 3. Never |  |
| 312 | Are the toilets clean?  N.B: **Clean** means no smile, no faecal material around pit on floor | 1. Always 2. Sometimes 3. Never |  |
| 313 | Is there Queuing for the latrine during your break | 1. Always 2. Sometimes 3. Never |  |
| 314 | Where do you usually execrate your faeces | 1. Home latrine 2. School latrine 3. Communal latrine 4. Open field |  |
| 315 | How Frequent you use a latrine | 1. Always 2. Mostly 3. Rarely |  |
| 316 | Where did you defecate yesterday? | 1. Open field(river, bush, backyard) 2. Home latrine 3. School latrine |  |
| **Hand washing behaviour** | | | |
| 401 | Do you know If you don’t wash your hands after going to toilet, could you get germs? | 1. Yes 2. No 3. Don’t know |  |
| 402 | Do you know When it is important to wash your hands? | 1. Yes  2. No  3. Don’t know | If 2 or 3 skip to 404 |
| 403 | If yes, when?  (Multiple response is possible) | 1. Washing after defecation 2. Washing before eating meals 3. Washing after eating meals |  |
| 404 | Do you know washing your hands with soap better than water only in diseases prevention | 1. Yes 2. No 3. Uncertain |  |
| 405 | Do you think hand washing is Important for disease prevention | 1. Yes 2. No 3. Uncertain |  |
| 406 | Do you think If people don’t wash their hands more often they will get sick | 1. Yes 2. No 3. Uncertain |  |
| 407 | Do you believe that washing hands just with water and soap is as good as washing hands with water? | 1. Yes 2. No 3. Uncertain |  |
| 408 | What do you think that children want to wash their hands? | 1. Conformity 2. Sensory benefits 3. Fun 4. Disease avoidance and 5. Getting better marks |  |
| 409 | Did you wash your hand | 1. Yes 2. No | If 2 skip to |
| 410 | When do you wash your hands (multiple response is possible) | 1. Before eating 2. After eating 3. After defecation |  |
| 411 | How frequent did you wash? | 1. Usually 2. Occasionally |  |
| 412 | Have you wash your hands yesterday | 1. Yes 2. No 3. Uncertain |  |
| 413 | How do you wash your hands (rearrange the following)  _______________________ | 1. Clean under your fingernails 2. Rinse your hands well with running water 3. Wet your hands with water and lather with a bar of soap 4. Rub your hands and scrub all surfaces up to your wrists 5. Dry them in the air 6. Continue for at least 30 seconds |  |
| 414 | What makes you to wash your hands  (multiple response is possible) | 1. Disgust 2. Fear 3. Comfort 4. Nurture 5. Status 6. Affiliation 7. Attraction |  |
| 415 | What Materials used for hand washing | 1. Soap/ash and water 2. Water only 3. Others, ___ | If 2 or 3 skip to 417 |
| 416 | At what junctures would you be washing your hands with soap? | 1. After defecation 2. Before eating 3. After eating |  |
| 417 | What makes you not to use soap | 1. Soap is not important 2. Unattractive soap 3. Unavailability of soap 4. Don’t know |  |
| 418 | Why not washing hands  (multiple response is possible) | 1. Not important 2. Forgetfulness 3. Laziness 4. Lack of time 5. Lack of clean water 6. Lack of soap 7. Don’t know |  |

**House hold based questionnaire (interview and observation)**

**Student Identification Code: ______________________,**

**Data collector: __________________________________,**

**Date of collection: _______________________________,**

| S. No | Questions | Possible answer | | | | skip |
| --- | --- | --- | --- | --- | --- | --- |
| **Water Supply facility** | | | | | | |
| 201 | What is the main source of drinking water | 1. Piped water into dwelling 2. Public tap or standpipe 3. Tube well or borehole 4. Rivers | | | |  |
| 202 | How long does it take you to go to your main water source, get water, and come back | 1. 30 minutes and less 2. More than 30 minutes 3. On premises 4. Don’t know | | | | If 3 or 4 skip to 205 |
| 203 | Who usually collects water? | 1. Adult women 2. School age female children 3. Adult men 4. School age male children | | | |  |
| 204 | Is there Queuing to fill container | 1. Yes 2. No | | | |  |
| 205 | Is water available all the time or for several hours a day? | 1. Yes 2. No 3. Don’t know | | | |  |
| 206 | Is water usually available during the following times? | 1. Morning , Yes/No 2. During the day, Yes/No 3. Evening, Yes/No | | | |  |
| 207 | Drinking Water storage | 1. Narrow necked water container 2. Wide water container | | | |  |
| 208 | Covered with lid  (during visit) | 1. Yes 2. No | | | |  |
| 209 | Separate cup next to container  (during visit) | 1. Yes 2. No | | | |  |
| **Latrine facility** | | | | | | |
| 301 | Do you have a latrine | | 1. Yes 2. No | | If 2 skip to 310 | |
| 302 | Is it first latrine? | | 1. Yes 2. No | |  | |
| 303 | Does latrine have | | Hole cover: Yes/No  If concrete slab: slant toward Hole: Yes/No  Roof: Yes/No  Wall: Yes/No  Door: Yes/No  Wastebasket: Yes/No | |  | |
| 304 | Are there obstacles in the path(dense vegetation , waste or debris in its path,  major crevices or holes, mud) | | 1. Yes 2. No | |  | |
| 305 | Depth from faeces to slab >50cm | | 1. Yes 2. No | |  | |
| 306 | What is the condition of the floors? | | 1. Solid and very clean 2. Cracking upswept 3. Dilapidated and dirty | |  | |
| 307 | Fresh urine on pit | | 1. Yes 2. No | |  | |
| 308 | Faces Inside Latrine Structure | | 1. Yes 2. No | |  | |
| 309 | Faces Outside Latrine Structure | | 1. Yes 2. No | |  | |
| 310 | faeces in compound | | 1. Yes 2. No | |  | |
| **Hand washing facility** | | | | | | |
| 401 | Is there hand washing facility | | | 1. Yes 2. No | | If 2 skip to 406 |
| 402 | What kind of facility | | | 1. Sink and faucet 2. Bucket 3. Basin 4. Tippy tap | |  |
| 403 | Is the facility near the latrines? | | | 1. Yes 2. No | |  |
| 404 | Is there water in the containers? | | | 1. Yes 2. No | |  |
| 405 | Is there soap, ash, or other near the wash stand? | | | 1. Yes 2. No | |  |
| 406 | Where family members wash their hands | | | 1. In the facility 2. Elsewhere in the compound | |  |
